# Exploration of Microbial Factories for Synthesis of Nanoparticles – A Sustainable Approach for Bioremediation of Environmental Contaminants

**DOI:** 10.3389/fmicb.2021.658294

**Published:** 2021-06-04

**Authors:** Riti T. Kapoor, Marcia R. Salvadori, Mohd Rafatullah, Masoom R. Siddiqui, Moonis A. Khan, Shareefa A. Alshareef

**Affiliations:** ^1^Amity Institute of Biotechnology, Amity University, Noida, India; ^2^Department of Microbiology, Biomedical Institute-II, University of São Paulo, São Paulo, Brazil; ^3^School of Industrial Technology, Universiti Sains Malaysia, Penang, Malaysia; ^4^Chemistry Department, College of Science, King Saud University, Riyadh, Saudi Arabia

**Keywords:** bioremediation, green synthesis, microbes, nanoparticles, wastewater treatment

## Abstract

The nanomaterials synthesis is an intensifying research field due to their wide applications. The high surface-to-volume ratio of nanoparticles and quick interaction capacity with different particles make them as an attractive tool in different areas. Conventional physical and chemical procedures for development of metal nanoparticles become outmoded due to extensive production method, energy expenditure and generation of toxic by-products which causes significant risks to the human health and environment. Hence, there is a growing requirement to search substitute, non-expensive, reliable, biocompatible and environmental friendly methods for development of nanoparticles. The nanoparticles synthesis by microorganisms has gained significant interest due to their potential to synthesize nanoparticles in various sizes, shape and composition with different physico-chemical properties. Microbes can be widely applied for nanoparticles production due to easy handling and processing, requirement of low-cost medium such as agro-wastes, simple scaling up, economic viability with the ability of adsorbing and reducing metal ions into nanoparticles through metabolic processes. Biogenic synthesis of nanoparticles offers clean, non-toxic, environmentally benign and sustainable approach in which renewable materials can be used for metal reduction and nanoparticle stabilization. Nanomaterials synthesized through microbes can be used as a pollution abatement tool as they also contain multiple functional groups that can easily target pollutants for efficient bioremediation and promotes environmental cleanup. The objective of the present review is to highlight the significance of micro-organisms like bacteria, actinomycetes, filamentous fungi, yeast, algae and viruses for nanoparticles synthesis and advantages of microbial approaches for elimination of heavy metals, dyes and wastewater treatment.

## Introduction

The environmental pollution is one of the major problems of society. Water is essential for life and industrial as well as economic growth of nation. The heavy metals, organic compounds, insecticides, fertilizers, industrial effluents, and sewage are the principal environmental contaminants. The disposal of contaminants in water streams and rivers leads to contamination of water resources and adverse effect on aquatic ecosystem (Sharma et al., 2015). The excessive application of synthetic dyes in different industrial activities and use of pesticides in crop fields is responsible for water and soil pollution (Choi et al., 2018; Doshi et al., 2018; Hu et al., 2018). The disposal of untreated effluent in water resources may be due to inefficiency of wastewater treatment plants against some specific pollutants present at low concentration degrade surface water quality and give adverse impact on ecosystem and health of human-beings (Ajaz et al., 2020; Oon et al., 2020). However, contaminants can be used as a resource to fabricate nanoparticles via biogenic route for pollutants deterioration (Huang et al., 2018; Seifan et al., 2018; Mandeep and Shukla, 2020). The environmental attenuation depends on various technologies such as adsorption, chemical reactions, photocatalysis and filtration for contaminants removal from environment. Conventional methods are bound with various limitations such as expensive, energy intensive, generation of hazardous toxic chemicals, requirement of high temperature and pressure and uneconomical method because of their inability to completely purify wastewater and no option to reuse the material (Sharma et al., 2015). Nanoparticles are increasingly applied for the wastewater treatment due to their large surface area, high reactivity and degree of functionalization (Goutam et al., 2018). The treatment of wastewater can be performed by using pure or mixed microbial culture due to the synergistic metabolic action. The periphyton biofilm can be applied for degradation of dyes (Shabbir et al., 2020). The removal of dyes has been reported through the utilization of genetically modified microbes (Kumar et al., 2020). Dong et al. (2019) found that *Enterococcus gallinarum* and *Streptomyces* S27 degraded azo dyes by azoreductase enzyme. Laccase showed significant degradation potential for many dyes (Iark et al., 2019). The laccase degrades dye by non-specific free radical mechanism to form phenolic compounds and there will be no formation of aromatic amines (Chivukula and Renganathan, 1995).

The characteristics and efficiency of nanotechnology-based materials makes them appropriate for treatment of environmental contaminants as they have improved catalysis, high surface area which reflects high activity (Dwevedi, 2019). The degradation of environmental pollutants is challenging with conventional methods due to the complex nature of mixture with less reactivity and more volatility. The recent researches have shown the application of nanomaterials for solving most of the issues related to water quality and its recycling (Uddandarao et al., 2019). The treatment of wastewater and industrial effluent based on nanotechnology can provide water with less toxic substances, heavy metals and other impurities (Zonaro et al., 2017; Salem and Fouda, 2020). El-Kassas et al. (2016) reported the various nanomaterials for the removal of inorganic and organic pollutants.

**FIGURE 1:**
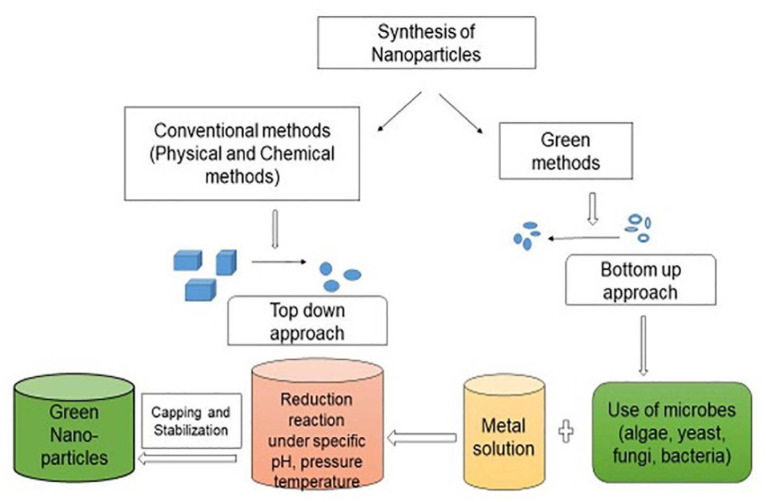
Synthesis of nanoparticles by different methods.

Nanoparticles have large surface area with surface energy which can easily absorb huge amount of pollutants. They can catalyze chemical reactions with fast speed with less energy intake as compared to huge material, thus check the release of contaminants. Due to the unique surface chemistry of nanomaterials as compared to conventional methods, can target contaminants with their functional groups for remediation. The desired alterations in shape, size, absorptive capacity and chemical components of nanomaterials enhances the performance of nanomaterials can provide significant benefit for treatment of contaminants (Sekoai et al., 2019; Wu et al., 2019). Application of biogenic substances will promote green technology as there will be very less generation of a sludge and it can offer a safe alternative for remediation of environmental pollutants. The interception of green chemistry and nanotechnology has paved a path to green nanotechnology (Anastas and Warner, 1998). The particles in the range from 1 to 100 nm are considered as nanoparticles which may present in an aggregate or free condition. Nanoparticles are basic component of nanotechnology as they are fundamental sources of various nanostructured devices. Nanoparticles are produced either by top-down or bottom-up methods as shown in Figure 1. Top-down approach is a process of conversion of large structures into small ones with the help of physical methods. The bottom-up approach utilizes small atoms or molecules to produce nanoparticles by self-assembly or supra-molecular chemistry. The biological mode of nanoparticle synthesis via bottom-up approach has been emerged as novel and green strategy. The biosynthesis of nanoparticles has got recognition due to less toxicity, biocompatibility, energy efficient and eco-friendly nature of the process with less sludge production hence can be used in pharmaceutical industry and biomedical applications (Mohammadlou et al., 2016; Ijaz et al., 2020; Noman et al., 2020; Goutam and Saxena, 2021). Micro-organisms in contaminated environment adapt and modify themselves for the degradation of xenobiotic compounds exhibiting the enormous catabolic activity toward the polluted environment (Hsueh et al., 2017). Biogenic synthesis of metal nanoparticles has received advantages due to their physico-chemical properties and wide applications in biotechnology (Slavin et al., 2017; Goutam et al., 2020). The global generation of metallic nanoparticles is estimated as 13.7 billon United States dollars and it is anticipated to increase 50 billon United States dollars by the year 2026. Main objective of the present review is to provide information on recent developments for the fabrication of functional nanoparticles, their characterization techniques and applications for remediation of pollutants.

## Factors That Affecting Nanoparticles Synthesis

Different experimental conditions such as pH, temperature, raw materials concentration, size and procedure can affect production and utilization of microbial nanoparticles (Baker et al., 2013). The pH of the medium affects microbial synthesis of nanoparticles. Reports have revealed that pH of solution medium affects size and composition of produced nanoparticles (Patra and Baek, 2014). The temperature is another parameter that influences nanoparticles synthesis in physical, chemical and biological methods. The physical method requires very high temperature (>350°C), whereas chemical methods require less than 350°C temperature. The production of nanoparticles by using green technology generally requires temperature less than 100°C (Rai et al., 2006). The pressure plays a pivotal role in nanoparticles synthesis as it can alter dimension and form of synthesized nanoparticles. Reduction of metal ions with biogenic sources was fast at ambient pressure condition (Tran et al., 2013). The synthesized biogenic nanoparticles quality is significantly affected by exposure period (Darroudi et al., 2011). The shape, size and adsorption capacity of the nanoparticles play a significant function in determination of the nanoparticle properties. The melting point of nanoparticles has been reported to decrease when the size of particles reduces to the nanometer scale. Baer et al. (2013) stated that appearance of nanoparticles influences their chemical properties. The change in the chemical properties has been observed when the individual nanoparticles come in contact with the surface of other nanoparticles. This process promotes the development of tuned nanoparticles. Environmental conditions play a pivotal role in synthesis of nanoparticles. Sarathy et al. (2008) reported conversion of single nanoparticle into core-shell nanoparticle by absorbing materials or reacting with other materials either by oxidation or corrosion. The expenses required for the synthesis of nanoparticles is also an important component. To promote potential applications of nanoparticles, expenses related with their production required to be controlled. Physical method requires expensive equipment whereas chemical method provides high yield in less time but it is expensive method (Ding et al., 2015). The nanoparticles production by biological methods involves low cost and can be applied at large scale (Gour and Jain, 2019).

## Microbial Synthesis of Nanoparticles

The green technology is an extensively accepted procedure for bioremediation due to clean, safe, non-toxic effect and environmentally benign method (Mishra et al., 2014; Salvadori, 2019). The production of nanoparticles by microbes is bottom up technique in which most of the reactions are reduction/oxidation. The basic concept of bioremediation is the change of harmful pollutants into less harmful compounds. Nanoparticles produced from microbes can transform pollutants into the compounds with less toxicity, solubility and mobility (Wasi et al., 2008). The metallic nanoparticles produced by biological methods are more stable at room temperature for long duration in comparison to metallic nanoparticles generated via chemical routes (Balakrishnan et al., 2017). The capping of the microbial proteins over the metallic nanoparticle surface provides stability to the biosynthetic procedures. The cost of production of the nanoparticles can be decreased to 1/10th in comparison to the chemical synthesis protocols by applying proper methods. Significant amount of contaminants can be removed with less number of biogenic nanoparticles. The biogenic nanoparticles show large surface area with high catalytic reactivity and they do not assemble due to the presence of capping agents released by microbes. The nanoparticles can be synthesized by microbes either intracellularly or extracellularly. Mishra et al. (2014) stated that extracellular biosynthesis is popular due to its low cost as it can be done without downstream processing. Microbes absorb precursor metal ions and can produce respective nanoparticles by using detoxification process (Mohseniazar et al., 2011). Microorganisms do not require high energy (Kumari et al., 2017) and there is no need to add capping or stabilizing agents thus application of microbes is cost effective process (Makarov et al., 2014). The synthesis of fine, uniform and functional nanoparticles under normal conditions is a challenging task (Tang et al., 2017). The biogenic sources provide a safe, cost-effective and environmentally benign method to fabricate metal nanoparticles (Ovais et al., 2018). The advantages such as well-defined morphologies, ease of production, scaling and enhanced biocompatibility have been lucrative for scientists to utilize biological resources as nanofactories (Singh et al., 2016). Microorganisms such as virus, fungi, yeast, algae, marine microbes, actinomycetes, bacteria have been widely used for the production of nanoparticles with gold, silver, copper, silicon, iron, nickel, cadmium and lead as described below and listed in Table 1.

**TABLE 1:** Biological synthesis of metal nanoparticles using different microbes.

**S. no.**	**Sources**	**Type of nanoparticles**	**Synthesis methods**	**Experimental conditions**	**Characterizations**	**Morphology**	**Size (nm)**	**References**
**Filamentous fungi**								
1.	*Aspergillus niger*	Silver	Extracellular	T: 25°C; t: 72 h	TEM, ESI	Spherical	20	Gade et al. (2008)
2.	*Fusarium solani*	Silver	Extracellular	T: 25°C; t: 24 h	TEM, FTIR	Spherical	5–35	Ingle et al. (2009)
3.	*Pleurotus sajor-caju*	Silver	Extracellular	T: 25°C; t: 72 h; pH 6	SEM	Spherical	5–50	Nithya and Ragunathan (2009)
4.	*Coriolus versicolor*	Silver	Extracellular Intracellular	T: Room; pH 10	FTIR, TEM, XRD	Spherical	25–75	Sanghi and Verma (2009)
5.	*Penicillium fellutanum*	Silver	Extracellular	T: 5°C; t: 24 h; pH 6	TEM	Globular	5–25	Kathiresan et al. (2009)
6.	*Trichoderma viride*	Silver	Extracellular	T: 27°C; t: 48 h; pH 7.2	TEM, FTIR	Spherical	5–40	Fayaz et al. (2010)
7.	*Epicoccum nigrum*	Silver	Extracellular	T: 55°C; pH 12	XRD, TEM	Spherical	1–22	Qian et al. (2013)
8.	*Guignardia mangiferae*	Silver	Extracellular	T: 25°C; t: 12 h	HR-TEM, SAED, XRD	Spherical	5–30	Balakumaran et al. (2015)
9.	*Fusarium oxysporum*	Silver	Extracellular	T: 50°C; pH 6	FTIR, TEM	Spherical	5–13	Husseiny et al. (2015)
10.	*Arthroderma fulvum*	Silver	Cell filtrate	T: 55°C; t: 12 h; pH 10	XRD, TEM	Spherical	15.5	Xue et al. (2016)
11.	*Colletotrichum* sp. ALF2-6	Silver	Cell free extract	T: 50–80°C; alkaline pH	FTIR, XRD, TEM	Myriad	5–60	Azmath et al. (2016)
12.	*Duddingtonia flagans*	Silver	Extracellular	T: 60°C; pH 10	DLS, TEM	Quasi-spherical	30–409	Costa Silva et al. (2017)
13.	*Fusarium oxysporum*	Silver	Cell-free filtrate	T: 28°C	DLS, SEM	Spherical	24	Hamedi et al. (2017)
14.	*Aspergillus oryzae* MTCC no. 1846	Silver	Cell filtrate	T: 90°C; pH 10	XRD, TEM, FTIR	Spherical	7–27	Phanjom and Ahmed (2017)
15.	*Fusarium keratoplasticum*	Silver	Culture filtrate	T: 35°C; t: 48 h	XRD, FTIR	Spherical	6–36	Mohmed et al. (2017)
16.	*Rhizopus stolonifera*	Gold	Culture filtrate	T: 40°C; 48 h	XRD, TEM, FTIR	Spherical	9.47	AbdelRahim et al. (2017)
17.	*Trichoderma longibrachaitum*	Silver	Culture filtrate	T: 28°C; t: 72 h	FTIR, TEM	Spherical	10	Elamawi et al. (2018)
18.	*Penicillium oxalicum* GRS-1	Silver	Extracellular	T: 60°C; pH 7	XRD, FESEM	Spherical	10–40	Rose et al. (2019)
19.	*Aspergillus fumigatus* BTCB10	Silver	Cell-free filtrate	T: 25°C; pH 6	ATR-FTIR, XRD, SEM	Spherical	322.8	Shahzad et al. (2019)
**Yeast**								
1.	*Candida glabrata*	CdS	Intra and extracellular	pH 3.5; 40,000 g	TEM	Hexamer	20 Å, 29 Å	Dameron et al. (1989)
2.	*Pichia jadinii*	Au	Intracellular	T: 28°C; t: 24 to 72 h	TEM	Various	–	Gericke and Pinches (2006)
3.	*Yarrowia lipolytica NCIM3589*	Au	Cell wall	T: 30°C; t: 72 h	SEM TEM	Particles and plates	15	Agnihotri et al. (2009)
4.	*Saccharomyces cerevisiae*	TiO_2_	Extracellular	T: 60°C, t: 10–20 min	X-ray, TEM	*Spherical*	12	Jha et al. (2009)
5.	*Saccharomyces cerevisiae*	Au	Cell wall cytoplasm	T: 15 min to 72 h	TEM	Spherical	15	Sen et al. (2011)
6.	*Candida albicans*	Au	Cell-free extract	T: 25°C; pH 7	TEM	Spherical	5	Ahmad et al. (2013)
7.	*Rhodotorula mucilaginosa*	Ni/NiO	*Extracellular*	T: 30°C; pH 4; t: 60 min	AFM, XPS, FTIR	*Spherical*	5.5	Salvadori et al. (2016)
8.	*Chlorococcum humicola*	Silver	Cell extract	T: Room	FTIR, TEM, SEM	Spherical	16	Jena et al. (2013)
9.	*Aphanothece* sp.	Silver	Cell extract	T: Room	EDX, SEM	Spherical	44–79	Sudha et al. (2013)
10.	*Sargassum muticum*	Silver	Cell extract	T: Room	FTIR, XRD, TEM	Spherical	5–15	Azizi et al. (2013)
11.	*Caulerpa racemosa*	Silver	Cell extract	T: Room	TEM, XRD	Spherical Triangular	5–25	Kathiraven et al. (2014)
12.	*Bifurcaria bifurcata*	Copper oxide	Cell extract	T: 100–120°C	FTIR, XRD, TEM	Crystalline	5–45	Abboud et al. (2014)
13.	*Sargassum longifolium*	Silver	Cell extract	T: Room	TEM, SEM	Spherical	40–85	Rajeshkumar et al. (2014)
14.	*Sargassum bovinum*	Palladium	Cell extract	T: 60°C	TEM, XRD, EDX	Octahedral	5–10	Momeni and Nabipour (2015)
15.	*Sargassum tenerrimum*	Gold	Cell extract	T: Room	HRTEM, FTIR	Rounded	5–45	Ramakrishna et al. (2016)
16.	*Sargassum ilicifolium*	Aluminum oxide	Cell extract	T: 25°C; t: 24 h; pH 4	TEM, SEM	Hexagon	35	Koopi and Buazar (2018)
**Bacteria**								
1.	*Lactobacillus* species	Titanium	Cell culture	pH 2–4	XRD, TEM	Spherical	40–60	Prasad et al. (2007)
2.	*Pseudomonas Putida* NCIM 2650	Silver	Extracellular	T: 37.5°C; t: 48 h; pH 6	FTIR, SEM	Spherical	70	Thamilselvi and Radha (2013)
3.	*Serratia nematodiphila*	Silver	Extracellular	T: 35°C; t: 24 h	TEM, XRD	Crystalline	10–31	Malarkodi et al. (2013)
4.	*Escherichia coli* (DH5a)	Silver	Extracellular	T: 15 min	TEM	Spherical	10–100	Ghorbani (2013)
5.	*Bacillus* strain CS11	Silver	Extracellular	T: Room	TEM	Globular	42–92	Das et al. (2014)
6.	*Bacillus methylotrophicus*	Silver	Extracellular	T: 28°C, t: 24 h	TEM, EDX	Spherical	10–30	Wang et al. (2016)
7.	*Novosphingobium* species	Silver	Extracellular	T: 25°C; t: 48 h	XRD, TEM	Spherical Crystalline	8–25	Du et al. (2016)
8.	*Pseudomonas fluorescens* CA 417	Silver	Extracellular	T: 80°C; pH 8	FTIR, XRD, EDS, TEM	Cubic Spherical Oval	10–60	Syed et al. (2016)
9.	*Bacillus amyloliquefaciens*	Titanium dioxide	Cell culture	T: 37°C; t: 72 h	FTIR, TEM, XRD	Spherical	22–97	Khan and Fulekar (2016)
10.	*Brevibacillus formosus*	Gold	Cell culture	T: 37°C; t: 24 h	FTIR, TEM, DLS	Spherical	5–12	Srinath et al. (2017)
11.	*Pseudomonas* sp. ef1	Silver	Cell culture	T: 22°C; t: 24 h	SEM, TEM, EDS	Spherical	50	John et al. (2020)
**Actinomycetes**								
1.	*Streptomyces hygroscopicus*	Gold	Intracellular	T: 35°C; 72 h	TEM	Spherical	10–20	Waghmare et al. (2014)
2.	*Streptomyces kasugaensis* NH28 strain	Silver	Cell filtrate	T: 27°C; t: 72 h	TEM, FTIR	Rounded	4.2–65	Składanowski et al. (2016)
3.	*Streptomyces capillispiralis*	Copper	Extracellular	T: 35°C; t: 6 h	TEM, XRD	Spherical	3.6–59	Hassan et al. (2018)
4.	*Streptomyces* species	Silver	Extracellular	T: 35°C; pH 8	TEM, FTIR	Spherical	2.3–85	El-Gamal et al. (2018)
**Marine microbes**								
1.	*Sargassum wightii*	Gold	Extracellular	T: 12 h	XRD, TEM	Spherical	8–12	Singaravelu et al. (2007)
2.	*Fucus vesiculosus*	Gold	Extracellular	pH 7; t: 8 h	XRD, TEM	Spherical	20–50	Mata et al. (2009)
3.	*Pichia capsulata*	Silver	Culture filtrate	pH 6; T: 5°C; t: 24 h	TEM	–	50–100	Manivannan et al. (2010)
4.	*Rhodosporidium diobovatum*	Lead	Intracellular	T: 25°C; t: 96 h	XRD	Spherical	2–5	Seshadri et al. (2011)
5.	*Gelidiella acerosa*	Silver	Extracellular	T: 48 h at 120 g AgNO_3_	SEM, XRD, TEM	Spherical	22	Vivek et al. (2011)
6.	*Ulva fasciata*	Silver	Extracellular	T: 100°C; t: 4 h	FTIR, SEM, TEM	Spherical	28–41	Rajesh et al. (2012)
**Virus**								
1.	Tobacco mosaic virus	CdS, PbS, SiO_2_, and Fe_2_O_3_	Surface	CdCl_2_ Pb (NO_3_)	TEM	Nano-tubes	10–40	Shenton et al. (1999)
2.	M13 bacteriophage	ZnS, CdS	Inorganic synthesis	T: 0–25°C; t: 24 h	HRTEM STEM	ND	560 × 20 nm quantum dot nano-wires	Mao et al. (2003)
3.	Tobacco mosaic virus	Gold	–	T: 20 min	TEM	Spherical	5	Kobayashi et al. (2012)

### Filamentous Fungi Mediated Synthesis of Metallic Nanoparticles

The filamentous fungi can be used as a potential source for the nanoparticles synthesis. The mycelium of fungi has high surface area which secretes huge amount of proteins that can participate directly in nanoparticles production (Mohanpuri et al., 2008). The production of nanoparticles by filamentous fungi is considered better due to their capacity to secrete proteins, enzymes and metabolites, simple scaling up and downstream handling, economic feasibility, increased surface area due to presence of mycelia and low-cost requirement for production procedures (Fouda et al., 2018; Spagnoletti et al., 2019). Different filamentous fungi species grow very fast and their maintenance at laboratory conditions is easy (Fouda et al., 2018). The nanoparticles fabrication with nanoscale dimension through fungi shows more monodispersity as compared with those synthesized by bacteria. *Fusarium oxysporum* in the presence of aqueous AuCl_4_^–^ ions with NADH-enzyme-mediated reaction releases reducing agents into the solution for the formation of gold nanoparticles. The synthesized nanoparticles show long-term stability due to the protein binding capacity by linkage of cysteine and lysine residues (Das et al., 2017). The filamentous fungi have high regenerative ability with environmental-benign production for synthesis of metal nanoparticles in significant amount with its commercial feasibility (Bansal et al., 2005). *Aureobasidium pullulans, Aspergillus niger, Cladosporium resinae, Penicillium* species, *Funalia trogii, Ganoderma lucidum, Rhizopus arrhizus* and *Trametes versicolor* absorbed heavy metals from polluted sites which was used for nanoparticles production (Say et al., 2003). Salvadori et al. (2013) reported uptake of Cu(II) by *Hypocrea lixii* dead biomass and production of copper nanoparticles. The same microorganism was able to produce NiO nanoparticles both extra and intracellularly (Salvadori et al., 2015).

*Fusarium oxysporum* exhibited extracellular synthesis of Au–Ag nanoparticles when treated with equimolar mixture of tetrachloroaurate ion and silver nitrate (Senapati et al., 2005) and in the presence of hexachloroplatinic acid it can produce platinum nanoparticles (Riddin et al., 2006). *Aspergillus flavus* synthesized silver nanoparticles (9 nm size) as it can reduce silver ions due to presence of sil genes in their plasmid (Vigneshwaran et al., 2007). Salvadori et al. (2014) stated that *Aspergillus aculeatus* dead biomass was reported to produce NiO nanoparticles (5.89 nm size) which were organized in form of film. Due to the presence of metabolites, fungi are better resource for synthesis of nanoparticles in comparison to bacteria (Singh et al., 2016). Zhang et al. (2011) found biosynthesis of gold nanoparticles in vacuoles of filamentous fungi and they also explained the functions of fungal proteins for capping of gold nanoparticles. Filamentous fungi are known as better candidate for metallic nanoparticles synthesis due to the presence of different enzymes in their cells and simple handling procedures (Khandel and Shahi, 2018). Filamentous fungi show metal uptake capacities and it can be easily cultured in huge amount by solid substrate fermentation. *Verticillium* species produced gold nanoparticles intracellularly after the exposure to chloroauric acid solution. Gericke and Pinches (2006) found the synthesis of gold nanoparticles by *Verticillium luteoalbum*. There was no effect of age on the shape of the gold nanoparticles but number of nanoparticles was reduced significantly with the use of old cells. The biomass of *Fusarium oxysporum* was used for generation of silver nanoparticles (Karbasian et al., 2008). Saxena et al. (2014) stated that genetic modification methods can be applied to enhance properties of nanoparticles.

### Yeast Mediated Synthesis of Metallic Nanoparticles

Most of the yeast genera can accumulate significant amount of heavy metals. The detoxification mechanism in yeast cells takes place by glutathione, metallothioneins and phytochelatins. Dameron et al. (1989) called yeast cells as semiconductor crystals or quantum semiconductor crystals as they have the ability to synthesize semiconductor nanoparticles such as cadmium and lead sulfide. *Pichia jadinii* synthesized gold nanoparticles in which gold ions were reduced by the enzymes present in cytoplasm or cell wall of yeast (Gericke and Pinches, 2006). The particles were not clumped together due to the peptide coating and exhibited very high stability as compared to nanoparticles synthesized by chemical methods. The quantum crystallites were produced by *Candida glabrata* and *Schizosaccharomyces pombe* when they were grown in cadmium salts (Williams et al., 1996). The gold nanoparticles were generated by *Yarrowia lipolytica* both extracellularly and intracellularly (Pimprikar et al., 2009). In *Yarrowia lipolytica*, nickel and cadmium in less concentration caused significant accumulation of metal-binding proteins (Strouhal et al., 2003). It not only resists heavy metals and also helps in hydrocarbons degradation (Bankar et al., 2009). *Yarrowia lipolytica* can be used for the synthesis of metallic nanoparticles as well as treatment of environmental contaminants such as heavy metals. The reduction of gold nanoparticles on the cell wall of dead cells of *Saccharomyces cerevisiae* significantly decreased the production cost of nanoparticles. The synthesis of cadmium nanoparticles by *Candida glabrata* and *Schizosaccharomyces pombe* has been reported by Dameron et al. (1989). *Rhodosporidium diobovatum* has been used for production of stable lead sulfide nanoparticles intracellularly (Seshadri et al., 2011). Soliman et al. (2018) described the synthesis of silver nanoparticles by *Candida albicans, Saccharomyces boulardii and Candida utilis.* Lian et al. (2019) reported use of *Magnusiomyces ingens* LHF1 for generation of stable selenium nanoparticles. Two patents were granted to Benedito Correa and his team members for synthesis of copper nanoparticles and use of fungal species for remediation of wastewater and industrial scale synthesis of copper nanoparticles was obtained in an inexpensive and eco-friendly manner. The patents were processes described to be used to bioremediate the copper tailings pond located at Sossego mine owned by the Company Vale SA, Canaã dos Carajás, Pará, Brazilian Amazonia region. Briefly, the processes consist of using the dead biomasses of the yeast *Rhodotorula mucilaginosa*, and of the filamentous fungi *Hypocrea lixii* and *Trichoderma koningiopsis* in aqueous solution containing copper, to removal of the metal, and concomitant synthesis of metallic copper nanoparticles, under conditions of physico-chemical parameters determined as optimal for carrying out the process. Obtaining as a final product, nanoparticles of metallic copper, in addition to promoting the uptake of the polluting transition metal (copper) from the impacted area. Being a low cost and ecofriendly process. The copper removal capacities and concomitant transformation into metallic copper NPs for *Rhodotorula mucilaginosa*, *Hypocrea lixii*, and *Trichoderma koningiopsis* were, respectively: 26.2 mg g^–1^, 19.0 mg g^–1^ and 21.1 mg g^–1^ (Correa et al., 2014a, b). Salvadori et al. (2016) explained the production of magnetic spherical nanoparticles of nickel or nickel oxide by dead organic matrix of *Rhodotorula mucilaginosa*. This technique can be of commercial importance due to the induction of magnetic metallic nanoparticles from liquid waste containing toxic metals which may results in detoxification of effluents and safe environmental release. Yeast mediated synthesis data of metallic nanoparticles are summarized in Table 1.

### Algae Mediated Synthesis of Metallic Nanoparticles

The algae are aquatic oxygenic photoautotrophs which can be used in production of nanoparticles (Castro et al., 2013). *Chlorella vulgaris*, *Dunaliella salina*, and *Nannochloropsis oculata* can produce silver nanoparticles of less than 15 nm size inside the cells within 48 h (Mohseniazar et al., 2011). The bio reduction ability of algae reflected significant potential in green synthesis of various metal oxide nanoparticles such as gold, silver, platinum, palladium, copper oxide and zinc oxide (Konishi et al., 2007; Xie et al., 2007; Oza et al., 2012; Momeni and Nabipour, 2015). Algae can produce complex inorganic nanomaterials both intracellularly and extracellularly (Sau and Murphy, 2004). The gold nanoparticles can be synthesized intracellularly in *Rhizoclonium fontinale* and extracellularly in *Lyngbya majuscula* and *Spirulina subsalsa* (Chakraborty et al., 2009). The metal ions can be attached to the cell surface via electrostatic interactions between ions and negatively charged carboxylate groups. Later, ions can be reduced by enzymes which lead to nuclei formation and grow with reduction of metal ions (Parial et al., 2012). Accumulation of gold (9–20 nm size) was reported in *Chlorella vulgaris* dried cell suspension (Hosea et al., 1986). *P. boryanum* in aqueous AuCl_3_ solution showed the deposition of gold (I)–sulfide nanoparticles at the cell wall. Cyanobacteria shows reduction of gold (III)–chloride to metallic gold with the formation of an intermediate Au (I) and gold (I)–sulfide (Lengke et al., 2006). Table 1 highlights the algae mediated synthesis of metallic nanoparticles.

### Bacteria Mediated Synthesis of Metallic Nanoparticles

The bacteria are known as potential bio-resources for generation of nanoparticles such as gold, silver, platinum, palladium, titanium, titanium dioxide, magnetite cadmium sulfide and others as listed in Table 1. Bacteria are important microbes for fabrication of nanoparticles due to their adaptability to adverse environmental conditions (Wang et al., 2017). Garole et al. (2018) reported that some bacteria were able to reduce or precipitate soluble toxic inorganic ions into nontoxic insoluble metal nanoparticles (Fang et al., 2019). Bacteria can form nanoparticles with metals and metalloids either intracellularly or extracellularly under different physico-chemical conditions like as exposure period, pH, temperature, concentration of bacteria and metal salts. The biomolecules present in the medium or cell wall components can reduce metal ions in an extracellular process. However, in intracellular process, by electrostatic interactions functional groups present on the cell wall attract metal and metalloids and metal ions interact with proteins present inside the cells for production of nanoparticles. Due to easy extraction procedure and high efficiency extracellular reduction appears to be more favorable as compared to intracellular reduction. Fang et al. (2019) stated that dead bacteria can be used for synthesis of nanoparticles same as live bacteria. Bacteria can be utilized as biocatalyst as they act as biological platform for mineralization (Iqtedar et al., 2019). The bacteria can mobilize or immobilize metals and can reduce or precipitate metal ions. Bacteria can catalyze different reactions due to their enzymes and can produce inorganic nanoparticles (Iravani, 2014). The large quantities of nanoparticles (100–200 nm size) can be produced in a pure form via extracellular secretion of bacterial enzymes. The metal binding capacity of the bacterial cells and S-layer make them useful for their applications in bioremediation. The cell wall of bacteria plays a very crucial function as metals may percolate into the cytoplasm via cell wall and transferred back to the wall for extracellular secretion. The cell wall with metal binding sites can be changed by chemical reactions for specific groups, such as amines and carboxyl groups, which converts positive charge into negative charge. Application of bacteria for nanoparticles production is lucrative process as it does not require any expensive and toxic chemicals for synthesis and stabilization procedures.

### Actinomycetes Mediated Synthesis of Metallic Nanoparticles

The actinomycetes show features of fungi and bacteria and they play a pivotal role in the production of metal nanoparticles. *Thermomonospora* species produced gold ions under harsh environmental conditions such as high temperature and alkaline conditions. *Rhodococcus* species alkali-tolerant actinomycetes induced for development of gold nanoparticles between 5 and 15 nm size (Ahmad et al., 2003). The high concentration of nanoparticles was reported on the cell wall in comparison to cell membrane. The metal ions were not toxic to the cells as there was no effect on cell growth after the fabrication of nanoparticles. Actinomycetes are good source for nanoparticles production due to their large surface area and with secretion of secondary metabolites. Actinomycetes can produce metallic nanoparticles either inside or outside the cells (Manivasagan et al., 2016; Hassan et al., 2018). Gold nanoparticles were synthesized by *Nocardia farcinica*, *Rhodococcus* sp*., Streptomyces viridogens, Streptomyces hygroscopicus, Thermoactinomyces* sp. and *Thermomonospora* species (Składanowski et al., 2016). The copper, zinc, manganese, and silver nanoparticles were also synthesized by using *Streptomyces* species (El-Gamal et al., 2018). Table 1 highlights the actinomycetes mediated synthesis of metallic nanoparticles.

### Marine Microbes Mediated Synthesis of Metallic Nanoparticles

The marine microbes have ability to synthesize nanoparticles as they exist in the bottom of sea and they are known to reduce huge amount of inorganic elements. Many marine microorganisms can produce metallic nanoparticles or mineral crystals with same properties of chemically synthesized nanomaterials. Marine microbes like bacteria (*Escherichia coli, Pseudomonas* species), cyanobacteria (*Spirulina platensis, Oscillatoria willei, Phormidium tenue*), yeast (*Pichia capsulata, Rhodospiridium diobovatum*), fungi (*Penicillium fellutanum, Aspergillus niger, Thraustochytrium* sp.) and algae (*Diadesmis gallica, Navicula atomus, Sargassum wightii, Fucus vesiculosus*) have been reported to produce inorganic nanoparticles (Kalabegishvili et al., 2012; Asmathunisha and Kathiresan, 2013). *Penicillium fellutanum*, marine fungus isolated from coastal mangrove sediment generates silver nanoparticles extracellularly after its exposure to silver nitrate (Kathiresan et al., 2009). Gomathi (2009) reported that marine fungi *Thraustochytrids* have poly-unsaturated fatty acids and show extracellular biosynthesis of composites of lipid and silver nanoparticles.

*Escherichia coli* AUCAS 112 and *Aspergillus niger* AUCAS 237 isolated from mangrove sediments reduced silver ions successfully and formed silver nanoparticles were monodispersed and globular in nature (Kathiresan et al., 2009). *Pseudomonas* sp. 591786 has also produced silver nanoparticles inside the cells which were polydispersed with various sizes from 20 to 100 nm (Muthukannan and Karuppiah, 2011). The culture filtrate of *Pichia capsulate*, a mangrove-derived yeast exhibited rapid production of silver nanoparticles (Manivannan et al., 2010). They revealed that protein was responsible for the silver nanoparticles production and it was present in the culture filtrate of yeast. *Rhodosporidium diobovatum* contained sulfur rich peptide which acted as a capping agent for the synthesis of lead sulfide nanoparticles (Seshadri et al., 2011). Govindaraju et al. (2008) reported the synthesis of silver, gold and bimetallic nanoparticles by *Spirulina platensis.* The protein secreted from *Oscillatoria willei*, marine cyanobacterium reduced silver ions and produced stable silver nanoparticles (Mubarak Ali et al., 2011a). Cadmium sulfide nanoparticles fabrication by applying C-phycoerythrin isolated from *Phormidium tenue* NTDM05 has been observed by Mubarak Ali et al. (2012). *Sargassum wightii*, brown seaweed was reported to synthesize gold nanoparticles (8–12 nm size) (Singaravelu et al., 2007). Mata et al. (2009) observed the use of *Fucus vesiculosus* for recovering gold from electronic scraps leachates and hydrometallurgical solutions for gold nanoparticles production as it has an ability of gold absorption and reduction. The extracellular production of silver nanoparticles in *Sargassum wightii* was reported by Govindaraju et al. (2009). Fucoidan, an algal polysaccharide application can stabilize gold nanoparticles and better method as compared to its synthetic production (Suwicha et al., 2010). Vivek et al. (2011) stated the production of silver nanoparticles by a red seaweed *Gelidiella acerosa*. Rajesh et al. (2012) reported that extracts of *Ulva fasciata* can be used as reducing agent for production of silver nanoparticles. *Navicula atomus* and *Diadesmis gallica* diatoms have the capacity to synthesize gold nanoparticles and silica-gold bio-nanocomposites (Kroger et al., 1999; Schrofel et al., 2011). Silicon-germanium nanocomposite was produced by *Stauroneis* species (Mubarak Ali et al., 2011b). *Pterocladia capillacae*, *Jania rubens, Ulva fasciata* and *Colpomenia sinuosa* were used for silver nanoparticles synthesis (Azizi et al., 2013). *Sargassum crassifolium*, seaweed, was used in gold nanoparticles production. *Cystoseira trinodis* synthesized CuO nanoparticles and reflected antioxidant potential and degraded methylene blue dye (Gu et al., 2018). Roni et al. (2015) used *Hypnea musciformis* seaweed as a stabilizing agent in synthesis of silver nanoparticles. Satapathy and Shukla (2017) observed the generation of highly monodispersed silver nanoparticles by marine cyanobacterium *Phormidium fragile*. The intracellular generation of gold nanoparticles was also reported by *Lyngbya majuscule* (Bakir et al., 2018). Marine microbes mediated synthesis data of metallic nanoparticles are summarized in Table 1.

### Virus Mediated Synthesis of Metallic Nanoparticles

The application of viruses in synthesis of nanoparticles is a good approach that has been capable to produce inorganic nanomaterials such as cadmium sulfide, silicon dioxide, iron oxide and zinc sulfide. Virus mediated synthesis data of metallic nanoparticles are summarized in Table 1. The problem in synthesis of inorganic nano-crystals has been observed in bacteria and fungi due to the use of protein framework, DNA recognizing linkers and surfactant assembled pathways. However, these restrictions can be solved by use of modified viruses with production of self-assemble surfaces with quantum dot structures with mono-disperse shape and size along with the length of nanoparticles. Zeng et al. (2013) reported the use of viruses for the production of quantum dots. The genetically modified tobacco mosaic virus generates nanoparticles which can change inorganic nano-crystals in three dimensional materials (Shenton et al., 1999). The synthesized viral films can be kept for long duration and can be stored for high-density engineered DNA with their application in pharmaceutical industry (Mao et al., 2003). Douglas et al. (2002) observed the use of cowpea chlorotic mottle virus and cowpea mosaic virus for the mineralization of inorganic materials. Tobacco mosaic virus helps in sulfide and crystalline nanowires mineralization (Shenton et al., 1999).

## Mechanism of Microbial Synthesis of Nanoparticles

Microorganisms can produce nanoparticles by the extracellular or intracellular enzymes as described below:

### Extracellular Enzymes

**FIGURE 2:**
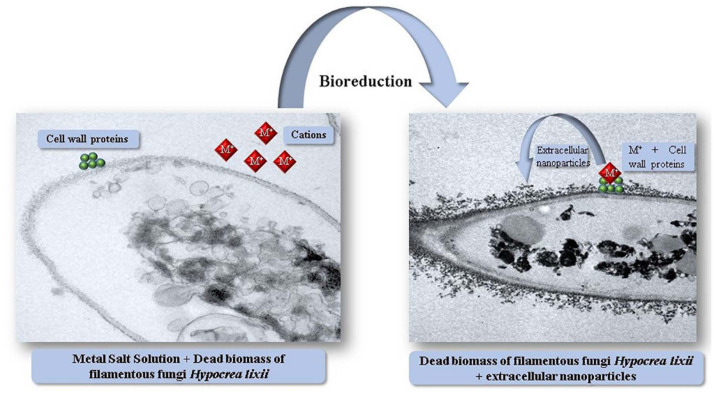
Illustration of the mechanism of extracellular synthesis of metal nanoparticles by dead biomass of *Hypocrea lixii.*

The extracellular microbial enzymes act as a reducing agent and play an important role in metallic nanoparticles production (Subbaiya et al., 2017). The extracellular enzymes such as acetyl xylan esterase, cellobiohydrolase D and glucosidase present in fungi takes part in synthesis of metallic nanoparticles (Ovais et al., 2018). *Rhodopseudomonas capsulata* produced gold nanoparticles extracellularly by electron transfer from NADH by NADH-reliant reductase enzymes. After accepting the electrons, gold ions reduced to form gold nanoparticles (He et al., 2007). *Fusarium oxysporum* was utilized as a reducing agent for gold and silver nanoparticles generation. The shuttle quinone and nitrate-reliant reductase obtained from *Fusarium* species were used in production of nanoparticles extracellularly (Senapati et al., 2005). *Fusarium semitectum* and *Fusarium solani* enzymes were used for extracellular production of silver nanoparticles (Ingle et al., 2009). *Cladosporium cladosporioides* and *Coriolus versicolor* were utilized for the extracellular synthesis of silver nanoparticles (Balaji et al., 2009). *Aspergillus fumigatus* extracellularly produced silver nanoparticles only in 10 min as compared to physical and chemical techniques (Bhainsa and D’Souza, 2006). *Sargassum wightii* reduced Au^3+^ ions to form gold nanoparticles (Singaravelu et al., 2007). *Chlorella vulgaris* synthesized gold nanoparticles (Lengke et al., 2006). The mechanism through which filamentous fungi synthesize nanoparticles extracellularly is not explained in the literature. Salvadori et al. (2013) reported interaction between metal ions and enzymes present in filamentous fungi cell wall and its successive reduction and nanoparticles production. Figure 2 shows a probable mechanism of extracellular nanoparticles synthesis by filamentous fungi.

### Intracellular Enzymes

**FIGURE 3:**
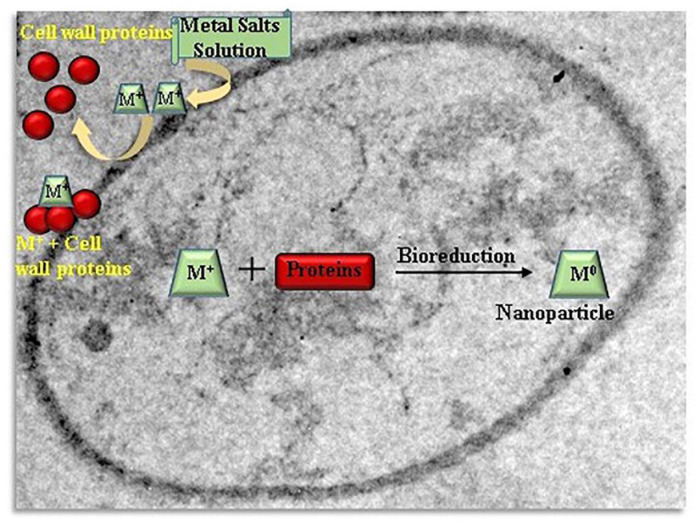
Schematic illustration of the mechanism of intracellularly nanoparticles formation by yeasts.

Actinomycetes such as *Rhodococcus* species and *Thermomonospora* species which were alkalo-tolerant and alkalo-thermophilic, respectively, were utilized for fabrication of gold nanoparticles intracellularly (Ahmad et al., 2003). Exposure of *Verticillium* species to an Ag^+^ ion solution showed intracellular reduction and fabrication of silver nanoparticles. Similar procedure was applied for production of gold nanoparticles by using *Verticillium* as a rich source of reducing enzymes (Ovais et al., 2018). Salvadori et al. (2017) suggested a probable natural procedure for the intracellular metal nanoparticles synthesis with yeasts. A possible mechanism behind the intracellular nanoparticles production is the electrostatic interaction between metal cations and amide groups present in yeast cell wall enzymes, reduction of the ions by enzymes which result in accumulation of metal ions and formation of nanoparticles. The Figure 3 schematizes a possible mechanism of intracellularly nanoparticles synthesis by yeasts.

## Application of Biogenic Nanoparticles for Bioremediation

Nano-bioremediation is an important branch of nanotechnology which deals with the removal of environmental contaminants such as organic and inorganic pollutants, dye, heavy metals from contaminated sites using nanoparticles synthesized from microbes. The nano-bioremediation is promising technology which provides eco-friendly, sustainable and feasible option for treatment of contaminants (Singh and Walker, 2006). As compared to the traditional methods, biosynthesized nanoparticles have some specific properties and can be utilized without any adverse effect in catalysis and degradation of organic pollutants (Naim et al., 2016). High efficiency of biogenic nanoparticles is due to vast surface area when particle size is decreased to nanoscale (Vanalakar et al., 2018).

### Degradation of Dyes

The biosynthesized nanoparticles show eminent catalytic activity because of the large surface area with significant number of active sites. Srivastava and Mukhopadhyay (2014) evaluated photocatalytic efficiency of biosynthesized SnO_2_-nanoparticles by *Erwinia herbicola* for dye degradation. The SnO_2_-nanoparticles reflected significant catalytic activity as 93, 94 and 98% deterioration of methylene blue, methyl orange and, erichrome black T, respectively, was observed. Bhargava et al. (2016) reported that surface proteins present on gold nanoparticles formed by *Cladosporium oxysporum* AJP03 enhanced adsorption of rhodamine B dye. *Pseudoalteromonas* species degraded Napthol Green B dye under anaerobic conditions (Cheng et al., 2019). *Pseudoalteromonas* species synthesized black colored iron-sulfur nanoparticles endogenously during degradation process which inhibited H_2_S release and metal sludge accumulation.

Copper nanoparticles were synthesized from *Escherichia* sp. SINT7 and they showed degradation of various azo dyes such as reactive black-5, congo red, direct blue-1 and malachite green (Mandeep and Shukla, 2020).

### Catalytic Dehalogenation

The chlorinated aromatic compounds are mostly utilized in various industrial applications due to their resistance against flame, oxidation and less water solubility. The excessive use of chlorinated aromatic compounds is responsible for water, soil and air pollution. Fang et al. (2019) reported the dehalogenation of aromatic compounds by biosynthesized Pd-based nanoparticles. The cell surfaces of *Desulfovibrio desulfuricans, Desulfovibrio vulgaris* and *Desulfovibrio* sp. “Oz-7” were used to produce palladium nanoparticles. The rate of dechlorination of biogenic palladium nanoparticles was thirty times higher as compared to chemical-Pd-nanoparticles. The modification in the catalytic activity was due to chemical composition and presence of functional groups on biogenic palladium-nanoparticles (Baxter-Plant et al., 2003). *Shewanella oneidensis* MR-1 produced palladium nanoparticles both intracellularly and extracellularly. The 4-nitrophenol, a nitro-aromatic contaminant present in dyes and synthetic pesticides adversely affects our central nervous system. It adversely affects our central nervous system. Zhang and Hu (2018) used *Bacillus* sp. GP to synthesize palladium and gold nanoparticles which showed the catalytic activity of Pd/Au nanoparticles for reduction of 4-nitrophenol. Cumbal et al. (2003) reported better affect of bio-Au-NPs/rGO on the reduction of 4-nitrophenol, but also high catalytic activity for the degradation of nitrobenzene. It was found that 72% catalytic activity was maintained after ten reduction cycles. Liou et al. (2007) stated iron nanoparticles can remediate carbon tetrachloride compound present in ground water. Studies revealed para-nitrophenol degradation to amino phenol in half an hour via gold nanoparticles synthesized by *Trichoderma viride* (Mishra et al., 2014).

### Removal of Heavy Metals Ions

Heavy metal pollution is a major environmental problem (Fujita et al., 2014). Wang et al. (2017) observed the reduction in the toxic impact of vanadium and chromium with *Shewanella loihica* PV-4 as removal efficiencies of V and Cr were 71 and 91%, respectively, after 27 days. Ha et al. (2016) used palladium nanoparticles for the elimination of hexavalent chromium from contaminated water. The biosynthesized palladium nanoparticles had small size with more surface-to-volume ratio in comparison to chemically reduced palladium nanoparticles so they showed better catalytic performance. Kimber et al. (2018) stated the intracellular synthesis of copper nanoparticles with *Shewanella oneidensis* MR-1. The silver nanoparticles synthesized from *Aspergillus niger* effectively decolorized 86% of dye within 24 h and dye was completely decolorized within 2 days. However, some limitations in biosynthesis of nanoparticles exist such as less production, contamination of biological cells and tough process of separation of nanoparticles from biological materials. There is an utmost need to search microbial diversity for new and sustainable microorganisms for biosynthesis of nanoparticles (Jiang et al., 2018). The iron oxide nanoparticles were synthesized from *Aspergillus tubingensis* and they were able to remove of heavy metals like lead 98%, nickel 96.45%, copper 92.19%, and zinc 93.99% from wastewater and reusability study revealed iron-nanoparticles had high regeneration ability up to five adsorption/desorption cycle (Mahanty et al., 2020).

Further researches should focus on the identification of the mechanism involved in synthesis of nanoparticles from biogenic sources and control of the morphology, size and dispersity.

## Conclusion and Future Perspectives

The bio resources such as microbes and microbial enzymes, if utilized successfully, can help in the biosynthesis of nanoparticles which can be proving as a potential game changer strategy. There is significant potential for microbial assisted metal nanoparticles synthesis as they are less toxic with high degradation capacity. It has also been discovered that the mechanism behind biologically synthesized nanoparticles is not well understood, despite the fact that stable nanoparticles can be generated by selecting suitable microorganisms and optimizing conditions. Thus, there is a need to choose suitable microbes or microbial consortia for large-scale sustainable production of nanoparticles. Microbial synthesis of nanoparticles can be achieved without the application of high temperature, pressure, energy, stabilizers and toxic chemicals. There is a need to synthesize nanoparticles with a wide range of organic functional groups by manipulating microbial enzymes for selective as well as multi-pollutants removal from wastewater. The nanoparticles synthesis via microbes such as bacteria, actinomycetes, fungi, yeast and algae has many advantages such as easy production, non-expensive, high efficiency, safe and eco-friendly approach. The microbial nanoparticles can be used at the contaminated sites for the treatment of pollutants. The residues left after the degradation of contaminants by microbial nanoparticles are biocompatible and can be separated easily by filtration or precipitation technique. The value-added products such as construction materials can be prepared with left residues by incorporating biochar, hence there will be zero waste at the end. Therefore, a greener route for nanoparticles synthesis opens new channels for many biotechnological applications. The biological synthesis of metallic nanoparticles has been achieved at laboratory scale but industrial scale escalation is needed for their mass production. The application of efficient microbes-assisted nanotechnology can boost the industrial economy but unfortunately only 1% nanotechnology materials have been commercialized till date. The cost effective production of microbial nanoparticles is required to make this process economically feasible and sustainable as per the requirements of industries. The cost-benefit analysis should also be conducted for its commercial exploitation as no cost related data is available till date. Application of chemicals, salts, reducing and stabilizing agents in chemical synthesis process are expensive whereas in microbial synthesis use of metal salts and media for microbial growth is also expensive. The waste biomass which is recyclable can be an alternative for the production of nanoparticles to lessen the expenditure. Further investigations about the biosynthetic pathways of microbes and researches in genetic engineering may open new avenues for breakthrough development of these promising nanofactories for scaling-up and industrial exploitation as efficient sustainable strategies for the bioremediation. Advanced computational tools are required to exploit the omics derived data for better understanding of microbial processes. Hence, green chemistry can be used successfully for production of nanoparticles by microbes and efforts in this direction will be a giant jump toward the adoption of green nanotechnology.

## Author Contributions

All the authors contributed equally to the study design, collection of data, development of the sampling, analyses, interpretation of results, and preparation of the manuscript. All authors have read and agreed to the published version of the manuscript.

## Conflict of Interest

The authors declare that the research was conducted in the absence of any commercial or financial relationships that could be construed as a potential conflict of interest.
